# Measurements agreement between low-cost and high-level handheld 3D scanners to scan the knee for designing a 3D printed knee brace

**DOI:** 10.1371/journal.pone.0190585

**Published:** 2018-01-10

**Authors:** Yoann Dessery, Jari Pallari

**Affiliations:** 1 Research & Development department, Peacocks Medical Group, Newcastle upon Tyne, United Kingdom; 2 Research & Development department, PODFO, Newcastle upon Tyne, United Kingdom; Northwestern University, UNITED STATES

## Abstract

Use of additive manufacturing is growing rapidly in the orthotics field. This technology allows orthotics to be designed directly on digital scans of limbs. However, little information is available about scanners and 3D scans. The aim of this study is to look at the agreement between manual measurements, high-level and low-cost handheld 3D scanners. We took two manual measurements and three 3D scans with each scanner from 14 lower limbs. The lower limbs were divided into 17 sections of 30mm each from 180mm above the mid-patella to 300mm below. Time to record and to process the three 3D scans for scanners methods were compared with Student t-test while Bland-Altman plots were used to study agreement between circumferences of each section from the three methods. The record time was 97s shorter with high-level scanner than with the low-cost (p = .02) while the process time was nine times quicker with the low-cost scanner (p < .01). An overestimation of 2.5mm was found in high-level scanner compared to manual measurement, but with a better repeatability between measurements. The low-cost scanner tended to overestimate the circumferences from 0.1% to 1.5%, overestimation being greater for smaller circumferences. In conclusion, 3D scanners provide more information about the shape of the lower limb, but the reliability depends on the 3D scanner and the size of the scanned segment. Low-cost scanners could be useful for clinicians because of the simple and fast process, but attention should be focused on accuracy, which depends on the scanned body segment.

## Introduction

The knee is the second most affected joint by osteoarthritis. Knee osteoarthritis has a world prevalence around 250 million people (3.8%), increasing with age [1,2]. Due to aging and increasing obesity, the prevalence of knee OA is expected to increase in developed countries in the next 20 years [2]. A knee brace is a non-pharmacological treatment for knee osteoarthritis recommended by Osteoarthritis Research Society International (OARSI) [3]. This medical device aims to relieve pain and delay knee surgery by unloading the affected compartment of the knee. However, poor compliance to this treatment is observed because of lack of effectiveness, discomfort, bad fit, migration of the brace, bulkiness, poor aesthetics, skin irritation, blisters and too much pressure on the knee [4–6]. These complaints are mainly due to the design and the fit of the knee brace, which could be solved with bespoke knee braces. Moreover, bespoke knee braces have been demonstrated to be more effective for knee osteoarthritis than off the shelf ones [7].

Additive manufacturing (AM), more popularly known as 3D printing, is a process of joining materials to make objects from 3D model data. AM is perfect to make well-designed/appealing lightweight bespoke products at no extra cost. Custom-made orthotics offer fit and comfort over off-the-shelf/mass-produced orthotics [7,8]. According to this and advantages of design freedom by AM, interest in AM to make bespoke orthotics devices has grown in the last decade [9]. Besides, 3D scans from the desired body part can be use to make orthotics with AM.

Manual measurement is the current reference method to take measurements in areas needing human anthropometry such as anthropometry survey, garment industry or orthotics and prosthetics industries. However, the manual method is time-consuming, relatively subjective as well as limited because of the use of only a few key measurements [10]. On garment industry impulse, 3D body surface scanners have had important technical developments in recent years. 3D body scanners capture highly accurate anthropometric data along with decreasing the duration of the process and providing less error in measurement compared with the traditional method [11]. Previous studies have shown that 3D scanning is an effective method to obtain anthropometric data of human body parts [12,13] and it may be beneficial in many fields to help with the designing and fabricating process [14–17].

3D scans have several advantages for orthotics compared to traditional techniques such as plaster casts, foam impressions or manual measurement. Firstly, they improve the experience of customers during their first visit by capturing the patient geometry in a precise (accuracy around 0.5-2mm), consistent, easy, clean and quick way [8,18–20]. Secondly, 3D scans avoid issues about storage space and data transfer, so the body part can be archived and used later if necessary. Finally, the scans data may then be used with Computer Assisted-Design software to process it (filtering, thickening, meshing,…) into an accurate 3D digital model which can be used to design the orthotic directly on the body part [20]. Then, finite-element analysis software allows to use this 3D digital model to evaluate the mechanical properties and effectiveness of an orthotic to optimize properties and functional performance, as by removing weight, moving pressure where needed [19,21–23].

Nevertheless, using 3D scans for orthotics is a new way and most of the studies have been done on foot [12,15,24,25]. Therefore, some limitations and questions are still pending, especially concerning the best position of the body part for the scan, the accuracy of the 3D scans and their use for other body parts [26,27]. Concerning the latter aspect, very accurate 3D body scanners are available on the market, but they are very expensive and not affordable for most orthotists. The last 5 years have seen development of low-cost handheld 3D scanners, which could be of help providing 3D scanners to each orthotist. Low-cost 3D scanners have been proven to be accurate in measuring human foot anthropometry [12,15], but shape and size of the lower limbs, such as the knee, cannot be compared to foot shape. Concerning lower limbs, only one study looked at differences between manual measurement and handheld 3D scanners [28]. This study found a good agreement between measurements from different sections of the lower limb, but they only used a high-level one and they did not look at the reliability of these measurements.

In the process of mass customization based on 3D printing, it is necessary to assess the low-cost 3D scanners for each body part. For now, only one 3D printed knee brace is currently on the market. However, the number of 3D printed knee braces is going to grow in coming years with the increase in knee problems and needs to delay surgery. Yet, no studies have looked at outcomes from handheld 3D scanners for knee scans. This study had two objectives. The first was to evaluate the agreement of measurements with a high level structured-light handheld 3D scanner and the technique currently used in orthotics, i.e. manual measurement with a tape measure. This would validate the use of 3D scanning to get knee measurements. The second objective was to analyze the agreement of measurements between a low-cost handheld 3D scanner and a high level one. This part was intended to assess the current capacity of low cost scanner for the purpose of knee brace mass customization.

## Materials and methods

### Participants

We enrolled 14 voluntary healthy participants with mean age 33.9 ± 11.2 years (95% Confidence Interval (CI): 27.4 − 30.4 years), height 1.76 ± 0.12m (CI: 1.69 − 1.82m), weight 77.8 ± 13.2kg (CI: 70.2 − 85.4kg), BMI 25.2 ± 3.8kg.m^-2^ (CI: 23.0 − 27.4 kg.m^-2^), leg length 92.2 ± 6.3cm (CI: 88.6 − 95.8cm). Orthopaedic/neurological conditions that may interfere with the testing position, i.e. not allowing to stand upright for 10 minutes, was the only exclusion criteria. This study was approved by the Peacocks Review Board and the protocol conforms to the principles of the Declaration of Helsinki. Oral informed consent was obtained from all participants.

### Equipment

We tested two different handheld scanners to scan the lower limbs: EVA Artec (*Artec Group*, *Luxembourg*, *Luxembourg*) and iSense (*3D Systems*, *Rock Hill*, *SC*, *USA*). The Eva scanner is a handheld high precision scanner used to scan full body or body segments. The iSense is a low-cost scanner to mount on an iPad and used to scan specific body segments. The characteristics of each scan are detailed in Table 1. The EVA scanner is powered by a wall outlet and interfaces with computer via USB2 and the Artec Studio software (v9.0, *Artec Group*, *Luxembourg*, *Luxembourg*). The scans are performed by turning around the subject without any need to specify the body segment scanned or any scanning parameters. The iSense scanner is mounted and plugged to an iPad2 and uses a free application to acquire scans: 3DSizeME (v2.0, *TechMed3D*, *Lévis*, *Qc*, *Canada*). A box is displayed on the screen to set depth, width, height and distance parameters of the box. This box has to fit the most possible the body segment scanned to improve the resolution of the 3D scan.

**Table 1:** 3D scanners characteristics.

Scanner	EVA (Artec)	iSense (3D Systems)
Acquisition software	Studio 9	3DSizeMe
Price	£13 700	£289+ iPad
Weight/Dimension	0.85kg / 262 x 158 x 63mm	0.099kg+iPad / 119.2 x 29 x 27.9mm
Cable	USB to PC + Power plug	USB to iPad
Scanning technology	Structured light	Infrared structured light
Working distance	From 0.4 to 1m	From 0.4 to 3.5m
3D resolution	0.5mm	0.9 (at 0.5m)– 30mm (at 3m)
Linear Field of View	214 x 148mm– 536 x 371mm	
Angular Field of View	30x21°	58x45°
Frame rate	16Hz	30Hz

It is important to specify that the accuracy of 3D scanners depends on both hardware and software, so both components are included when we speak about low-cost or high level scanner.

### Protocol

The participant was instructed to stand in an upright position with shoulder width feet position and maintain this steady posture for the whole measurement duration to guarantee accuracy. Measurement of the preferred leg of participants were taken with three methods: manual measurement, 3D scan with EVA scanner, 3D scan with iSense. The preferred leg was identified as the kicking leg. The same assessor successively performed all the measurements in same environment conditions to make sure of the validity of the comparison for each participant and the assessor was the same for all participants.

For manual measurement, the assessor measured the circumferences of the leg with a standard flexible tape measure (sensitivity 1mm). Measurements were taken with an interval of 30mm starting from the centre of the patella. Six and ten measurements were, respectively, taken above and below the mid-patella as well as the circumference at mid-patella. The measurement of each circumference was repeated twice.

Concerning scanning, participants stood on a 30cm-step to facilitate the scanning process. The assessor took three scans from the ankle malleolus to the top of the thigh with each scanner. We set iSense scanning parameters individually to ensure the best resolution for each participant. The post-processing of each scan was done with the Artec Studio software (version 9, *Artec Group*, *Luxembourg*, *Luxembourg*) and MSoft (version 2, *TechMed3D*, *Lévis*, *Qc*, *Canada*) for EVA and iSense, respectively.

**Fig 1:**
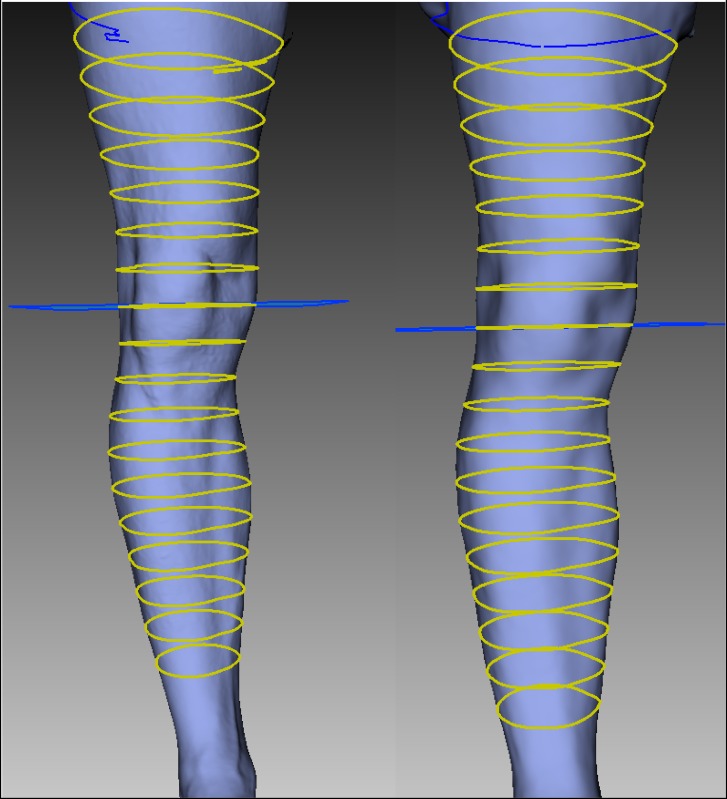
Lower limb sections. Example of the 17 sections on an EVA (left) and an iSense (right) 3D scan.

Each post-processed scan for a participant was saved as a ‘STL’ file and imported in Artec Studio software. All the scans were aligned to each other with the “Align” tools from Artec Studio software. Then, sections were created on each scan to have leg circumferences (Fig 1). Measurements sections were the same as previously described for the manual measurement (mid-patella, six above and ten below mid-patella). Mid-patella circumference is the start point and noted 0. Sections above mid-patella are identified as positive distance from the mid-patella: from +30mm to +180mm whereas sections below are negative: from -30mm to -300mm.

We compared the scanners in three ways: time to record the three scans, time for the post-processing of the three scans and leg’s circumference at each section in mm. For both time measurements, we started the stopwatch at the first action for the first scan and it was running until the end of the last action for the third scan. The times for manual measurement were not recorded because there was no post-processing and no way to have a 3D model. The manual measurement was used as an indicative measurement because it is the technique that is currently used in clinics while the EVA scanner was used because it is considered to be the reference scanner due to its resolution of 0.5mm with accuracy of 0.01mm (manufacturer’s technical information).

### Statistical analysis

Statistical analysis was conducted using Matlab (version 2016b, *The MathWorks*, *Inc*., *MA*, *USA*). We ran a Kolmogorov-Smirnov test to verify if the time data were normally distributed. Then, a Student t-test was used to compare the time to record and the time to process the three scans with each scanner. We set the level of significance at p < .05.

Concerning method comparison, we used the Bland-Altman method to assess the agreement between the observed values with each measurement method [29–31]. This well-established analysis is used to quantify agreement between two quantitative measurements. Bland-Altman method consists of computing first the differences between methods, then calculating the mean bias and 95% limits of agreement (LoA) of the differences.

We looked at the global agreement between methods to take measurements by using all the sections together with the non-constant and repeated measurement for each subject analysis described by Bland & Altman [29]. Then, we inspected agreement between methods for each section by using the constant and repeated measurement for each subject analysis [29]. For both analyses, we reported the absolute and relative bias and 95% LoA [32] and the 95% CI of bias and LoA, thus giving the precision of the estimates (SE; [33]). We also investigated the intraclass correlation coefficient (ICC) and the r value for uniformity of the bias [30] in the global analysis. Concerning the by-section analysis, we studied the coefficient of repeatability of each method [30]. ICC estimates and their 95% confident intervals were calculated based on a mean-rating (k = 3), absolute agreement, 2-way mixed-effects model (ICC(3,3)) [34,35]. The ICC is unitless and gives information about the reliability of each method. The uniformity of the bias permitted the study of the relationship between the bias and the magnitude of the circumferences. If a relationship was found, non-uniform bias and LoA were calculated and the equation of the bias was specified. The coefficient of repeatability estimates variation in repeated measurements made on the same subject under identical conditions and also indicates a baseline to judge between-method variability, with a lower value indicating better repeatability. The ICC and the uniformity of the bias were not estimated in the by-section analysis because of the poor degree of freedom (13) while the coefficient of repeatability was not relevant in the global analysis in regards to the disparities in circumferences between sections.

In regards to results from previous studies on 3D scanner [12,13,15,36,37], we set the clinical acceptable difference [38] between methods to ±1%.

## Results

### Manual vs. EVA scanner: Global assessment

The global assessment included 236 mean circumferences instead of 238 (17 sections x 14 participants) because we were not able to calculate EVA 180mm sections for one participant and -300mm sections for another participant.

**Fig 2:**
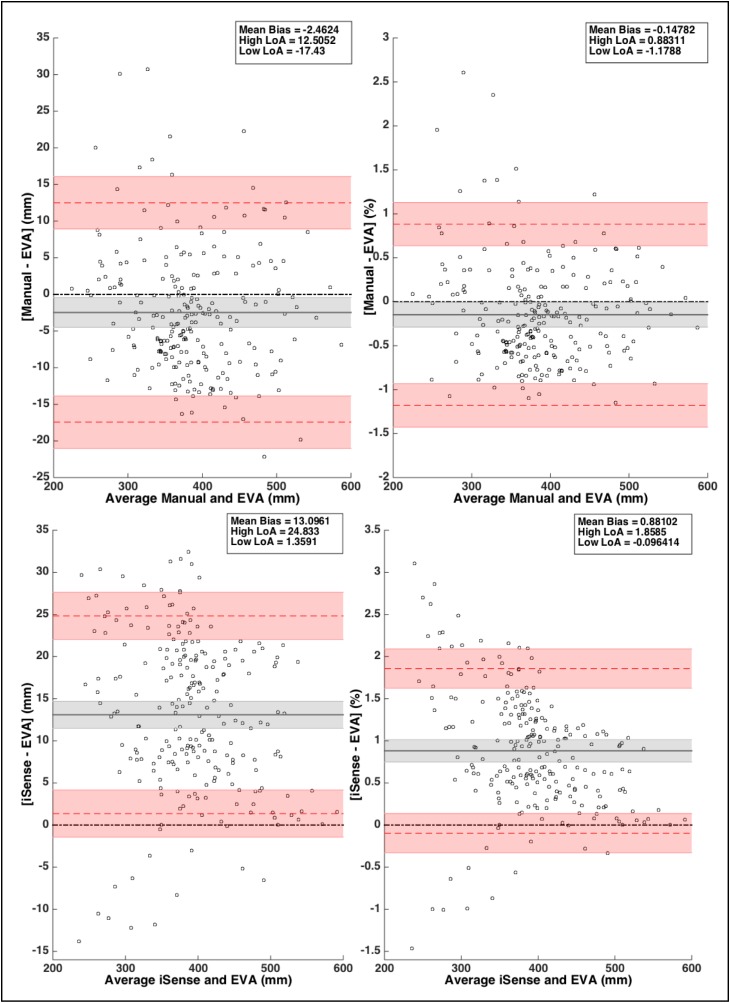
Uniform agreement between measurement methods. Bland-Altman plots illustrating the differences in the circumference as measured manually versus EVA scanner (A) and with iSense scanner vs EVA scanner (B). The left panels are absolute data and the right panels are relative data. Plots present differences between the two methods (mm or %) compared to average circumferences (mm) of the two methods. The black solid lines represent the mean bias of the differences with grey shaded areas being the 95% confidence interval of the bias, red dashed lines are the high and low limits of agreement surrounded by red shaded areas as 95% confidence interval.

The Bland-Altman plots (Fig 2A) illustrate absolute and relative agreements between the two methods. A negative bias indicates that measurements from EVA scanner overestimate the value in respect to manual measurements. From visual inspection and uniformity of the bias (r = -0.12), it did not seem that there was a relationship between bias and magnitude of circumferences, so a constant bias was used. The bias was -2.5mm (-0.15%) with LoA from -17.4 to 12.5mm (-1.2% to 0.9%). The SE was 2.0mm (0.14%) and 3.6mm (0.25%) for the bias and the LoA, respectively. The ICC between methods was 1 (95%CI = 0.99 − 1.0; p<0.001).

### Manual vs. EVA scanner: By-section assessment

Each section analysis was composed of 14 mean circumferences (one for each participant), except for 180mm and -300mm sections where only 13 mean circumferences were available.

The mean biases for the sections varied between -8.3mm and 3.4mm (-2.3% and 1.0%; Table 2). The biggest differences were observed in the 30mm, -60mm, -90mm and -120mm with relative mean differences being inferior to -1.5%. The estimates of biases and LoA were less precise in the extremities sections (Table 2). Except for the 60mm section, the coefficients of repeatability were higher for the manual measurement than for the EVA scanner, over 100% for section -60mm, -90mm, -150mm and -300mm.

**Table 2:** Agreement between manual measurements and EVA scanner. Comparing the absolute and relative means of the two manual measurements (N = 14) and means of the three scan measurements (N = 14), limits of agreement, coefficient of repeatability within methods and uniformity of the data for each section of the lower limb going from 300mm below the mid-patella (0mm) to 180mm above the mid-patella.

Manual vs. EVA							
Sections (mm)	Aver. circ. in mm	Abs. mean diff. in mm (SE)	Rel. mean diff. in % (SE)	95% Limit of Agreement (Mean ± 1.96SD)			Repeatability Manual/EVA
				Abs. Lower − Abs. Upper (mm)	Rel. Lower − Rel. Upper (%)	Abs. SE (mm)/Rel. SE (%)	
180	517.0	-4.2 (2.6)	-0.8 (0.5)	-22.5 − 14.1	-4.5 − 2.8	18.8/0.7	6.5/6.0
150	493.1	-1.9 (3.1)	-0.4 (0.6)	-24.6 − 20.9	-5.1 − 4.3	27.8/1.2	7.2/5.3
120	464.2	0.3 (2.9)	0.0 (0.7)	-21.3 − 21.8	-4.7 − 4.8	24.4/1.2	9.1/4.8
90	434.6	1.7 (2.3)	-0.4 (0.5)	-18.3 − 15.0	-4.3 − 3.5	13.9/0.8	8.5/5.3
60	411.5	-4.0 (1.8)	-1.1 (0.4)	-16.9 − 8.9	-4.1 − 2.2	7.8/0.5	5.4/7.1
30	398.3	-7.6 (1.7)	-1.9 (0.4)	-19.7 − 4.5	-5.0 − 1.2	6.7/0.4	7.6/5.6
0	386.1	-4.3 (1.5)	-1.1 (0.4)	-15.4 − 6.8	-4.0 − 1.8	5.2/0.4	8.3/5.9
-30	363.0	-2.7 (1.4)	-0.8 (0.4)	-12.7 − 7.3	-3.7 − 2.1	4.8/0.4	5.7/3.6
-60	353.1	-6.8 (1.8)	-1.9 (0.5)	-19.6 − 6.1	-5.5 − 1.6	8.0/0.6	8.4/3.0
-90	364.2	-8.3 (1.5)	-2.3 (0.4)	-19.4 − 2.8	-5.3 − 0.8	5.4/0.4	9.3/3.5
-120	377.6	-7.9 (1.2)	-2.1 (0.3)	-16.9 − 1.2	-4.4 − 0.2	3.7/0.2	6.3/3.8
-150	382.1	-3.6 (1.3)	-0.9 (0.3)	-12.8 − 5.7	-3.4 − 1.5	3.6/0.3	7.9/3.8
-180	371.2	0.2 (1.8)	0.0 (0.5)	-13.0 − 13.3	-3.6 − 3.6	8.4/0.6	7.2/4.7
-210	347.2	3.4 (2.4)	1.0 (0.7)	-13.9 − 20.7	-4.0 − 5.9	15.5/1.3	7.2/5.2
-240	316.9	2.8 (2.9)	0.9 (0.9)	-18.5 − 24.1	-5.9 − 7.6	24.1/2.4	7.7/5.3
-270	287.8	2.9 (3.1)	1.0 (1.1)	-19.7 − 25.5	-6.7 − 8.7	27.6/3.2	6.9/5.0
-300	268.1	2.1 (2.5)	0.9 (0.9)	-14.1 − 18.4	-5.2 − 7.0	16.4/2.4	10.2/4.8

Aver. Average; circ., circumference; abs., absolute; diff., difference; SE, 95% confidence interval; rel., relative. A negative bias or limits of agreement indicates that measurements from EVA scanner overestimates the value in respect to manual measurements.

### iSense vs. EVA: Timing

The EVA scanner collected three scans in a shorter time than the iSense scanner (410s ± 118s vs. 507s ± 94s, respectively; p = .02). On the other hand, the time to process the scan and export STL files was longer with the EVA scanner (3588s ± 423s) than with iSense scanner (460s ± 169s; p <.001). Thus, the mean time to make one scan was 137s for the EVA and 169s for the iSense while the mean time to process one scan was 1196s for the EVA and 153s for the iSense.

### iSense vs. EVA: Global assessment

**Fig 3:**
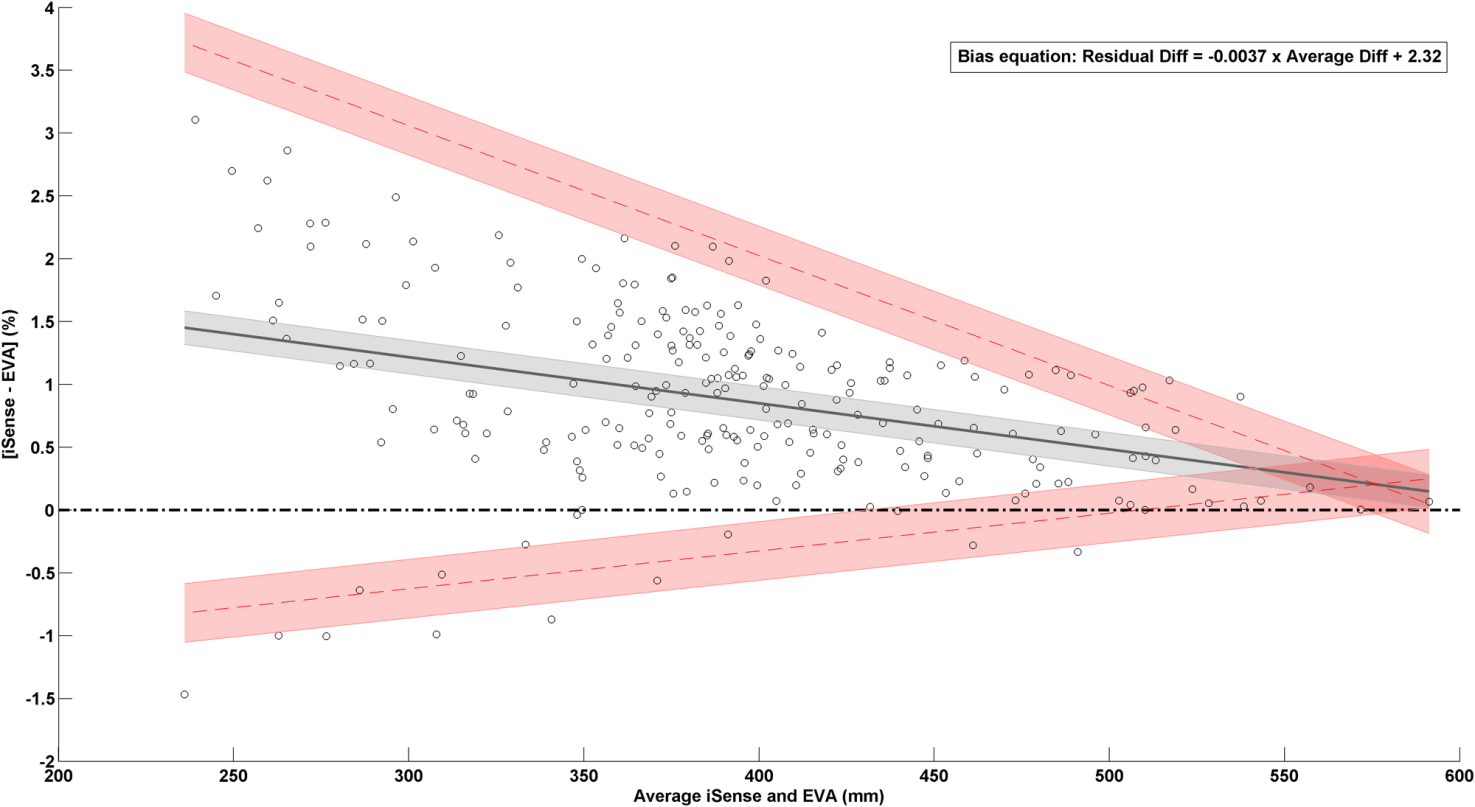
Non-uniform agreement between EVA and iSense scanners. Bland-Altman plots illustrating the relative differences (%) in the circumferences as measured with iSense scanner vs EVA scanner compared to average circumferences (mm) of the two methods. The black solid line represents the non-uniform mean bias of the differences with the grey shaded area being the 95% confidence interval of the bias, red dashed lines are the high and low limits of agreement surrounded by red shaded areas as 95% confidence interval. The legend indicates the equation of the non-uniform bias.

The Bland-Altman plots (Fig 2B) show absolute and relative agreements between both scanners. A positive bias indicates that measurements from iSense scanner overestimates the value in respect to EVA measurements. A constant bias of 13.1mm (0.88%) with LoA from from 1.4 to 24.8mm (-0.1% to 1.86%). The SE was 1.6mm (0.13%) and 2.8mm (0.23%) for the bias and the LoA, respectively. The ICC between methods was 0.99 (95%CI = 0.7 − 1.0; p<0.001). From visual inspection and uniformity of the bias (r = -0.18) of the absolute data, the relationship between bias and magnitude of circumferences was small. On the other hand, the Bland-Altman plot with relative data (Fig 2B; right panel) showed a stronger relationship between bias and circumferences (r = -0.36). The Fig 3 shows that the residual bias was going from 1.5% for lower circumferences to 0.1% for bigger circumferences with LoA decreasing with bigger circumferences.

### iSense vs EVA: By-section assessment

The mean biases for the sections varied between 9.1mm and 19.6mm (1.9% and 5.6%; Table 3). The biggest differences were observed in the -60mm, -90mm, -120mm, -270mm and -330mm with relative mean differences superior to 4%. The estimates of biases and LoA were less accurate in the lower extremity sections (Table 3). The coefficients of repeatability were higher for the iSense scanner than for the EVA scanner for all the sections: up to 50% from 0mm to 180mm sections and over 100% for sections located under the mid-patella.

**Table 3:** Agreement between iSense and EVA scanners. Comparing the absolute and relative means of the three iSense scan measurements (N = 14) and means of the three EVA scan measurements (N = 14), limits of agreement, coefficient of repeatability within methods and uniformity of the data for each section of the lower limb going from 300mm below the mid-patella (0mm) to 180mm above the mid-patella.

iSense vs. EVA							
Sections (mm)	Aver. circ. in mm	Abs. mean diff. in mm (SE)	Rel. mean diff. in % (SE)	95% Limit of Agreement (Mean ± 1.96SD)			Repeatability Manual/EVA
				Abs. Lower − Abs. Upper (mm)	Rel. Lower − Rel. Upper (%)	Abs. SE (mm)/Rel. SE (%)	
180	517.0	-4.2 (2.6)	-0.8 (0.5)	-22.5 − 14.1	-4.5 − 2.8	18.8/0.7	6.5/6.0
150	493.1	-1.9 (3.1)	-0.4 (0.6)	-24.6 − 20.9	-5.1 − 4.3	27.8/1.2	7.2/5.3
120	464.2	0.3 (2.9)	0.0 (0.7)	-21.3 − 21.8	-4.7 − 4.8	24.4/1.2	9.1/4.8
90	434.6	1.7 (2.3)	-0.4 (0.5)	-18.3 − 15.0	-4.3 − 3.5	13.9/0.8	8.5/5.3
60	411.5	-4.0 (1.8)	-1.1 (0.4)	-16.9 − 8.9	-4.1 − 2.2	7.8/0.5	5.4/7.1
30	398.3	-7.6 (1.7)	-1.9 (0.4)	-19.7 − 4.5	-5.0 − 1.2	6.7/0.4	7.6/5.6
0	386.1	-4.3 (1.5)	-1.1 (0.4)	-15.4 − 6.8	-4.0 − 1.8	5.2/0.4	8.3/5.9
-30	363.0	-2.7 (1.4)	-0.8 (0.4)	-12.7 − 7.3	-3.7 − 2.1	4.8/0.4	5.7/3.6
-60	353.1	-6.8 (1.8)	-1.9 (0.5)	-19.6 − 6.1	-5.5 − 1.6	8.0/0.6	8.4/3.0
-90	364.2	-8.3 (1.5)	-2.3 (0.4)	-19.4 − 2.8	-5.3 − 0.8	5.4/0.4	9.3/3.5
-120	377.6	-7.9 (1.2)	-2.1 (0.3)	-16.9 − 1.2	-4.4 − 0.2	3.7/0.2	6.3/3.8
-150	382.1	-3.6 (1.3)	-0.9 (0.3)	-12.8 − 5.7	-3.4 − 1.5	3.6/0.3	7.9/3.8
-180	371.2	0.2 (1.8)	0.0 (0.5)	-13.0 − 13.3	-3.6 − 3.6	8.4/0.6	7.2/4.7
-210	347.2	3.4 (2.4)	1.0 (0.7)	-13.9 − 20.7	-4.0 − 5.9	15.5/1.3	7.2/5.2
-240	316.9	2.8 (2.9)	0.9 (0.9)	-18.5 − 24.1	-5.9 − 7.6	24.1/2.4	7.7/5.3
-270	287.8	2.9 (3.1)	1.0 (1.1)	-19.7 − 25.5	-6.7 − 8.7	27.6/3.2	6.9/5.0
-300	268.1	2.1 (2.5)	0.9 (0.9)	-14.1 − 18.4	-5.2 − 7.0	16.4/2.4	10.2/4.8

Aver., average; circ., circumference; abs., absolute; diff., difference; SE, 95% confidence interval; rel., relative; iSS, iSense. A positive bias or limits of agreement indicates that measurements from iSense scanner overestimates the value in respect to EVA scanner.

## Discussion

Manual measurement is currently the reference method to take lower limb measurements for knee braces. This method is simple, inexpensive and accurate, but the inter- and intra-reliability is poor and information about leg shape is limited [39]. Our study showed that a high-level 3D scanner provides lower limb measurements with similar accuracy, but better repeatability. In the objective of mass customization, a low-cost 3D scanner enables the generation of a usable scan 9 times more quickly than a high level 3D scanner, however it overestimates the lower limb circumferences, especially for small circumferences.

With the growth of AM technology for orthotics and prosthetics [40], handheld 3D scanners are going to be an essential tool for clinicians. These scanners allow clinicians to easily and quickly capture the needed body part. The outcome can then be used to perfectly fit the medical device on the body part and improve the properties of the medical device [20,41]. Previous studies demonstrated accuracy of the handheld 3D scanners for foot shape to design 3D printed foot orthoses [18–20,24,25], but nobody has looked at lower limb shape for knee braces. Our study showed that high level handheld 3D scanners could be used to capture lower limb’s shape in order to make a bespoke product. High-level scanners demonstrated excellent agreement (ICC: 0.99 − 1.0) and 0.15% mean differences with the currently used technique of measurement for orthotics (manual measurement). These results are consistent with Cau et al.’s study [28], which compared lower limb circumferences between manual measurements and handheld 3D scanner in normal and obese populations. Besides, the high-level 3D scanner has a better intra-assessor reliability. More importantly, while orthotists usually take three to nine manual measurements to order a custom-made knee brace, the 3D scan has the advantage of giving the real shape of the lower limb to design the knee brace.

Several studies have compared high-level 3D scanner outcomes to manual measurement for different body parts circumferences [13,36,37,42]. However, clinicians cannot afford current reference 3D scanners because of the high cost. And yet, only a few studies have looked at low-cost handheld 3D scanners and those focused on foot shape [12,15]. Taha et al. [15] are the only ones comparing the low-cost scanner to another method (manual measurement with measuring tape). With differences between 0.22% and 4.26% according to the measurements of the foot (length, width and circumferences), they concluded that low-cost scanner was accurate in measuring human foot anthropometry. Given that high-level scanners were previously revealed to be better than manual measurement, our study focused on comparison between low-cost and high-level scanners. We observed that the low-cost scanner overestimates the geometry of the leg by a mean bias of 13mm (0.88%) in a relatively consistent way (SE = 0.13%) and with comparable repeatability of the measurement than manual measurement. This difference is smaller than in Taha et al.’s study [15] and slightly higher to relative differences found in circumferences with high-level scanners compared to manual measurement. Indeed, Ifflaender et al. [36] indicated a mean difference of 0.45% in head circumference while 0.1% to 0.5% differences were found in hip and thigh circumferences by Jaeschke et al. [13] and -0.6% to 1.1% in thigh, knee and calf by Simenko and Cuk [37]. According to our defined clinically acceptable difference, data from the low-cost scanner seems to have a good agreement with high-level scanners. Nevertheless, a 13mm absolute difference seems considerable when we attempt to design a perfect fitting orthotics, but in certain case, padding on the medical device can counteract this difference. Therefore, according to the use, the user can decide if this absolute difference is acceptable or not. This use-dependent suggestion is confirmed by our result on the uniformity of the bias.

To our knowledge, our study is the first to be interested in the uniformity of the bias when using a 3D scanner. Thus, we demonstrated a relationship between agreement and the magnitude of the circumferences for the low-cost scanner, with higher circumferences having better agreement. This original finding means that the low-cost scanner may be more relevant for larger body parts, i.e. chest, hip or thigh rather than foot or calf. The intra-assessor reliability seems to behave in the same way with lower reliability for the smaller sections. This finding is very interesting because it is likely due to the software that is used to scan, and more particularly the reconstruction algorithm. Therefore, it would be possible to improve it to increase agreement between 3D scanners.

We are also the first study to look at the difference in timing to scan and post-process data with a low-cost scanner compared to a high-level scanner. Our scanning time of around 2min10s to scan the leg is consistent with timing from previous literature, relative to the body part scanned. Salles et al. [20] could scan as fast as up to 14 seconds, but no data are available on the mean time. Using full-body scans, recent studies acquired full-body in 4 to 10minutes [13,43]. While acquiring data is lightly quicker with the high-level 3D scanner (32s less/scan), our results revealed that the low-cost handheld 3D scanner used with 3DSizeMe software allows clinicians to have processed leg shape almost 9 times faster than with the high-level 3D scanner. The small difference in the acquisition time was likely due to automatic reset of the acquisition parameters (box) after each scan. Thus, we needed to set them again for each scan with the low-cost scanner. However, the software company could easily improve this part of the scan process. By contrast, the clinician or the brace manufacturer saves a lot of time getting the 3D leg shape model by using the low-cost 3D scanner and its software. The difference may be the result of the size of the files as well as the way data is captured.

Our study has several limitations. Firstly, the poor sample size limits the Bland-Altman by-section analysis. However, it does not influence the global assessment because we are considering sections as independent measurements. This global assessment gives us sufficient information about agreement between methods. The by-section analysis is supplementary information about the repeatability of measurements within each method, in which the low number of participants is less of an issue. Secondly, the way to set box parameters in 3DSizeMe software for iSense scanner was subjective and dependent on each participant. Because the scan resolution is dependent on the size of the box, the resolution is likely better for shorter limbs. Nevertheless, we controlled this limitation by having the box always be defined by the same assessor while being as close as possible to the area being measured. Finally, even if we are aware that both hardware and software can influence the outcomes of the scanning process, we considered scanning solutions as a package (software and hardware) in our study. While EVA Artec scanner is intended to be used with the Artec Studio software, low-cost scanners could be used with several apps. Because we suggested that overestimation of the leg shape with the low-cost scanner may have been due to the software, we could have compared results from other apps. Nevertheless, the purpose of this study was to compare a high-level scanner solution with a low-cost scanner solution for the first time. The choice of the scanner and software was based on their availability and their ease of use. However, it could be interesting to compare several low-cost scanners coupled with several software packages to look at the influence of each on the quality of the scans in future studies.

It is expected that the orthotists will use 3D handheld surface body scanners on a daily basis in the coming years because AM technology in the fields of orthotics and prosthetics is developing exponentially. For now, some orthotists are still reluctant to use this technology for two main reasons. The first is the high cost of high level 3D scanners and the lack of information about the accuracy and reliability of low-cost scanners. The second reason concerns the change in shape of the limb when a device is applied. With manual measurement and casting, orthotists may capture the change in shape of the body segment whereas 3D scanners only give surface topology. Thus, it is necessary to provide information to clinicians about both the advantages and limitations of devices to help them improve their knowledge as well as patients’ treatment. Our study aimed to bring more knowledge about 3D scanners agreement, accuracy and reliability compared to the currently used technique. We demonstrate that 3D scanners provide more information about the shape of the lower limb with, at least, as good intra-rater reliability as manual measurements. However, this depends on the 3D scanner. Low-cost 3D scanners’ intra-rater reliability is as good as manual measurement and quick post-processing, but with bias regarding the lower limb’s geometry. Concerning the change in shape, future research could examine the possibility of doing two scans: one with the normal shape and one with a tool constraining the flesh of the limb, e.g. a tubular net. This could benefit to the design of the 3D printed knee brace.
