# Correction: Measurements agreement between low-cost and high-level handheld 3D scanners to scan the knee for designing a 3D printed knee brace

**DOI:** 10.1371/journal.pone.0196183

**Published:** 2018-04-17

**Authors:** Yoann Dessery, Jari Pallari

Table 3 is incorrect. Please see the corrected Table 3 here.

**Table 3:** Agreement between iSense and EVA scanners. Comparing the absolute and relative means of the three iSense scan measurements (N = 14) and means of the three EVA scan measurements (N = 14), limits of agreement, coefficient of repeatability within methods and uniformity of the data for each section of the lower limb going from 300mm below the mid-patella (0mm) to 180mm above the mid-patella.

iSense vs EVA							
Sections (mm)	Aver. circ. in mm	Abs. mean diff. in mm (SE)	Rel. mean diff. in % (SE)	95% Limit of Agreement (Mean ± 1.96SD)			Repeatability iSS/EVA
				Abs. Lower ‒ Abs. Upper (mm)	Rel. Lower ‒ Rel. Upper (%)	Abs. SE (mm)/Rel. SE (%)	
180	523.9	10.2 (2.6)	2.0 (0.5)	-8.2 ‒ 28.6	-1.6 ‒ 5.5	18.1/0.7	8.6/6.0
150	498.4	9.3 (2.4)	1.9 (0.5)	-8.3 ‒ 26.8	-1.7 ‒ 5.5	15.5/0.7	7.8/5.3
120	468.4	9.1 (2.3)	2.0 (0.5)	-7.7 ‒ 25.9	-1.7 ‒ 5.6	14.3/0.7	7.3/4.8
90	440.7	10.6 (2.0)	2.5 (0.5)	-4.3 ‒ 25.5	-1.0 ‒ 5.9	10.7/0.6	7.4/5.3
60	420.5	13.4 (1.9)	3.2 (0.5)	-0.6 ‒ 27.3	-0.3 ‒ 6.7	8.4/0.5	8.4/7.1
30	409.2	13.5 (1.9)	3.3 (0.5)	-0.5 ‒ 27.5	-0.2 ‒ 6.9	9.1/0.6	7.9/5.6
0	394.2	11.4 (2.1)	2.9 (0.5)	-4.3 ‒ 27.0	-1.1 ‒ 6.9	11.5/0.7	8.7/5.9
-30	371.2	13.5 (2.1)	3.7 (0.6)	-2.0 ‒ 29.1	-0.5 ‒ 7.8	12.1/0.9	7.7/3.6
-60	365.9	18.4 (2.1)	5.0 (0.6)	3.0 ‒ 33.8	0.8 ‒ 9.2	11.4/0.9	9.0/3.0
-90	378.4	19.6 (2.4)	5.2 (0.6)	2.3 ‒ 36.8	0.6 ‒ 9.7	14.4/1.0	9.9/3.5
-120	390.6	17.8 (2.3)	4.6 (0.6)	1.3 ‒ 34.4	0.3 ‒ 8.8	13.2/0.9	9.6/3.8
-150	391.3	14.7 (2.2)	3.8 (0.6)	-1.5 ‒ 31.0	-0.5 ‒ 8.1	12.8/0.9	9.3/3.8
-180	377.1	12.2 (2.7)	3.3 (0.8)	-7.3 ‒ 31.6	-2.2 ‒ 8.7	18.8/1.5	9.9/4.7
-210	350.7	10.9 (3.1)	3.2 (0.9)	-11.5 ‒ 33.3	-3.6 ‒ 10.0	25.5/2.4	10.2/5.2
-240	320.8	11.2 (3.3)	3.6 (1.1)	-13.3 ‒ 35.7	-4.5 ‒ 11.7	30.8/3.4	10.8/5.3
-270	292.4	12.6 (3.4)	4.4 (1.3)	-12.7 ‒ 37.8	-4.8 ‒ 13.6	32.7/4.4	11.3/5.0
-300	267.8	14.7 (3.7)	5.6 (1.5)	-12.6 ‒ 41.9	-5.3 ‒ 16.4	38.6/6.2	11.6/4.5

Aver., average; circ., circumference; abs., absolute; diff., difference; SE, 95% confidence interval; rel., relative; iSS, iSense

A positive bias or limits of agreement indicates that measurements from iSense scanner overestimates the value in respect to EVA scanner.
